# Correction to “Advanced Urologic Cancer Consensus Conference (AUC3) 2025: Expert consensus on the management of renal cell and urinary tract cancers”

**DOI:** 10.3322/caac.70070

**Published:** 2026-01-30

McKay RR, Pal S, Xie W, et al. Advanced Urologic Cancer Consensus Conference (AUC3) 2025: expert consensus on the management of renal cell and urinary tract cancers. *CA Cancer J Clin*. 2026;e70052. doi:10.3322/caac.70052

Please note that Oleksandr N. Kryvenko’s middle initial was omitted in the originally published version of this article.

We apologize for this error.

